# Morphologic assessment of peritalar compensation in patients with advanced varus ankle osteoarthritis

**DOI:** 10.1007/s00256-026-05236-w

**Published:** 2026-05-02

**Authors:** Zachary J. Eatough, Rich J. Lisonbee, Andrew C. Peterson, Shireen Y. Elhabian, Megan K. Mills, Nicola Krähenbühl, Amy L. Lenz

**Affiliations:** 1 Department of Orthopaedics, University of Utah, 590 Wakara Way, Salt Lake City, UT 84108 USA; 2 Department of Mechanical Engineering, University of Utah, 1495 E 100 S, Salt Lake City, UT 84112 USA; 3 Kahlert School of Computing, University of Utah, 50 S Central Campus Dr, Salt Lake City, UT 84112 USA; 4 Scientific Computing and Imaging Institute, University of Utah, 72 S Central Campus, Salt Lake City, UT 84112 USA; 5 Department of Radiology, University of Utah, 50 N Medical Dr, Salt Lake City, UT 84132 USA; 6 Department of Orthopaedics, University of Basel, Spitalstrasse 21, 4031 Basel, Switzerland

**Keywords:** Varus ankle osteoarthritis, Weight-bearing computed tomography, Statistical shape modeling, Hindfoot alignment

## Abstract

**Objective:**

Varus ankle malalignment is observed in most ankle osteoarthritis patients with approximately half of these patients presenting with peritalar compensation, where the subtalar joint is aligned valgus to compensate for a varus tibiotalar joint. This study developed a 3D weight-bearing computed tomography–based multi-bone statistical shape model to quantify morphologic and alignment differences between compensated and non-compensated presentations of advanced varus ankle osteoarthritis.

**Materials and methods:**

Our assessment included 70 individuals, 44 diagnosed with advanced varus ankle osteoarthritis, and 26 asymptomatic controls. Each participant underwent weight-bearing computed tomography. Semi-automatic segmentations produced patient-specific 3D bone reconstructions of the distal tibia, distal fibula, talus, calcaneus, navicular, and cuboid. A multi-bone statistical shape model was created using each of the 3D bone reconstructions. Joint space distance, coverage area, and congruence index were measured at equivalent anatomic locations within articular coverage obtained from the statistical shape model.

**Results:**

Eleven principal component analysis modes retained 85.8% variance. Significant differences existed in mode 1 (medial malleolus and talar dome morphology, fibular positioning; 26.6% variance, *p* < 0.001 for all comparisons) and mode 3 (talar head morphology, midtarsal joint orientation; 10.8% variance, *p* < 0.05). Morphometric analysis showed 67.5% combined shape-alignment differences in non-compensated versus controls, predominantly affecting peritalar structures.

**Conclusion:**

Patients with non-compensated varus ankle osteoarthritis demonstrate decreased medial tibiotalar joint space, increased talofibular joint space, and increased peritalar joint coverage compared to compensated and asymptomatic ankles. These differences are driven primarily by alignment variation in non-compensated ankles. Complexity between these two clinical presentations should be taken into consideration with rehabilitation efforts and surgical planning.

## Introduction

Patients with advanced ankle osteoarthritis (OA) present with limited physical function, severe pain, and diminished quality of life [1]. Most ankle OA arises from prior trauma and leads to hindfoot deformities [2, 3]. Varus ankle malalignment occurs in approximately 55% of ankle OA patients, with approximately half showing peritalar compensation, where the subtalar joint is aligned valgus to compensate for a varus tibiotalar joint [2, 4]. This compensatory mechanism is well established [4–10]. Compensation may occur as a result of changes in bone shape and orientation or ligamentous length changes [4, 9]. Peritalar interactions among the subtalar, talonavicular, and calcaneocuboid joints engender a multifactorial approach when treating varus ankle OA [6, 11, 12].

Weight-bearing computed tomography (WBCT) enables accurate three-dimensional (3D) bone visualization and analyses of degenerative progression including joint space narrowing, osteophyte characterization, and subchondral pathologies [13–17]. The key advantage of WBCT over standard CT is that it captures the ankle under normal load, providing accurate bone alignment and position while minimizing errors from positioning or radiographic distortion [18, 19].

Statistical shape models (SSM) built from WBCT images allow comparison of bone shape and alignment between groups [20]. Combined with 3D joint measures, including articular coverage, congruence index, and joint space distance, SSM enables detailed group comparisons [20, 21]. Nelson et al. found that ankle shape was associated with injury history, suggesting that morphology may both change after injury and predispose individuals to it, potentially playing a key role in ankle OA development [22].

Prior SSM studies of the ankle and hindfoot have largely focused on characterizing asymptomatic anatomic variation and joint morphology [20, 21]. These investigations have provided important insight into osseous shape variability but have not examined peritalar morphology in the setting of advanced varus ankle OA or considered the role of hindfoot alignment as a compensatory mechanism. The 3D structural differences between compensated and non-compensated varus ankle deformities remain incompletely understood. A detailed assessment of peritalar bone morphology for these alignment patterns may provide insight into mechanisms of disease progression and targets for surgical or biomechanical intervention.. We hypothesize that [1] morphology and alignment will differ between compensated and non-compensated ankles and that these differences will affect midtarsal joint orientation, [2] joint space distance and articular coverage will be decreased within the patients with OA compared to the asymptomatic control group, and [3] patients with varus ankle OA compensation will have greater joint space width in the anterior-medial facet of the subtalar joint, less talonavicular joint coverage, and greater talofibular congruence index compared to patients in the non-compensated ankle OA group.

## Materials and methods

### Data source and study population

This retrospective cohort study included 27 patients with compensated varus ankle OA, 17 patients with non-compensated varus ankle OA, and 26 asymptomatic controls (previously evaluated in prior characterization studies) with no history of foot and ankle trauma, surgery, or pain [20, 21]. Participants were recruited to participate with Institutional Review Board approval in accordance with the Declaration of Helsinki from January 2017 to September 2023. Each of the enrolled participants’ feet was screened and examined by both a board-certified orthopedic surgeon (12+ years of experience) and a musculoskeletal radiologist (13+ years of experience) in a consistent manner to ensure each foot and ankle met inclusion criteria. Asymptomatic individuals aged between 40 and 70 years without a history of ankle trauma, any operative procedures, or chronic pain at the level of the foot and ankle served as controls. Varus OA inclusion criteria involved patients diagnosed with post-traumatic advanced ankle osteoarthritis such as distal tibial fracture (distal one-third of the tibial length, extending from the tibial plafond proximally to the metaphyseal-diaphyseal junction (AO/OTA Classification 43,44)) or chronic ankle instability due to recurring ankle sprains. Varus OA exclusion criteria consisted of patients with a history of a tibia shaft (diaphyseal) (AO/OTA Classification 41,42), talus fracture, osteotomy of the proximal/distal tibia, or any arthrodesis/osteotomy of the hind- and midfoot. Patients with neurologic diseases were not considered.

### Imaging and measurements

Each participant underwent a clinical WBCT scan from one of three of the authors’ institutions: University of Utah (CurveBeam PedCAT; 0.37 × 0.37 × 0.37 mm voxels), University of Basel (Siemens Healthineers system; 0.35 × 0.35 × 0.35 mm voxels), or Kantonsspital Baselland (Planmed Verity; 0.40 × 0.40 × 0.40 mm voxels). WBCT images were semi-automatically segmented via user-selected seed points which then followed an atlas-based, threshold-guided segmentation algorithm to generate 3D bone reconstructions of the distal tibia, distal fibula, talus, calcaneus, navicular, and cuboid (DISIOR Bonelogic Ortho Foot and Ankle 2.1, Helsinki, Finland). Segmentations were then verified and then revised as needed by the first author, who has over 5 years of experience in image segmentation, to ensure an accurate representation of osteophytes and bone-on-bone joint degeneration (Mimics 24.0, Materialise, Leuven, Belgium). Bone reconstructions were consistently smoothed and meshed using an adaptive remesh with 0.5-mm edge length to preserve relevant anatomic features while minimizing noise-related artifacts, then aligned via an iterative closest point protocol [23, 24]. A standard cutting plane was determined and applied from the shortest tibia/fibula pair (3-Matic 16.0, Materialise, Leuven, Belgium). Semi-automatic measurements determined which group a patient with OA would be included in (DISIOR Bonelogic Ortho Foot and Ankle 2.1, Helsinki, Finland). Varus ankle OA patients were required to have a measured talar tilt (TT) ≥ 1.9°, and were separated based on hindfoot alignment angle (HAA), where participants with a HAA < 12.5° were included in the compensated group and those with a HAA ≥ 12.5° were included in the non-compensated group [8].

### Morphometric analysis

A multi-bone (i.e., multi-domain) SSM was created using the distal tibia, distal fibula, talus, calcaneus, navicular, and cuboid 3D bone reconstructions to report shape variations between groups (ShapeWorks, 6.3.1) [25, 26]. Morphometric analysis included utilizing component scores from a principal component analysis (PCA) to compare participant contributions of retained modes of variation identified via parallel analysis [27, 28]. This was followed by a comparison of the correspondence particle (anatomically equivalent surface points distributed across all shapes) positions in two different coordinate reference frames which sought to separate shape and alignment differences. Shape was statistically analyzed with each bone’s correspondence models centered and aligned at the origin following a Procrustes analysis. Alignment was statistically analyzed with each multi-bone correspondence model centered and aligned together retaining its native WBCT alignment.

### Joint measurement analysis

Correspondence models from the multi-bone SSM were used to quantify morphometric differences between groups as well as to evaluate joint space measurements [29]. Joint space distance, congruence index, and coverage area were calculated and analyzed for the tibiotalar, tibiofibular, talofibular, subtalar, talonavicular, and calcaneocuboid joints for all participants (MATLAB, R2023a, MathWorks). Joint space distance was calculated using the nearest Euclidean distance between two opposing bone surfaces. Joint congruence index was determined utilizing mean and Gaussian curvature data from two opposing bone surfaces, where a congruence index of 0 mm^−1^ indicates a perfect matching of the two surface curvatures; as the index increases, the congruence of the two surfaces decreases [30]. Joint coverage area was measured by summing the area of faces with intersecting normal vectors with opposing bone surfaces for each bone within a respective joint.

### Statistical analyses

Each statistical test was conducted within MATLAB (*α* = 0.05). Utilizing the correspondence models, a Hotelling’s *T*^2^ test was used to compare bone shape and alignment differences between groups [31–33]. A Shapiro-Wilk test for normality was performed on the HAA and TT digitally reconstructed radiograph measurements, as well as correspondence particles within coverage for joint congruence index and joint space distance between groups [34]. For data that did not follow a parametric distribution, a Kruskal-Wallis non-parametric analysis was performed followed by a Dunn post hoc analysis [35, 36]. Parametric data was compared using a one-way ANOVA followed by a Tukey-Kramer post hoc analysis [37]. Intra joint coverage results were compared using pairwise tests which included either a paired *t-*test or Wilcoxon signed rank test for parametric and non-parametric distributions respectively.

## Results

### Demographics and measurements

**Fig. 1:**
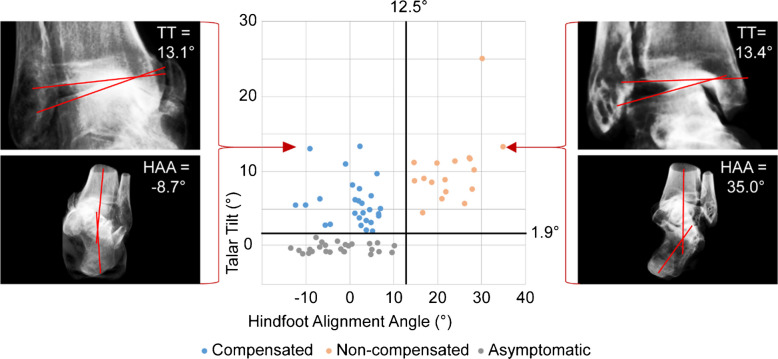
Scatter plot illustrating the relationship between hindfoot alignment angle (HAA) and talar tilt (TT) across three groups: non-compensated, compensated, and asymptomatic individuals. Gray points represent 26 asymptomatic individuals. Blue points represent 27 patients with a varus tibiotalar tilt (TT > 1.9°) but neutral or valgus hindfoot alignment (HAA < 12.5°). Orange points represent 17 patients with a varus tibiotalar tilt (tilt > 1.9°) and varus hindfoot alignment (HAA > 12.5°). Specific patients from both OA groups are shown. Compensated patient (sex: M, age: 61, BMI: 25, etiology: instability). Non-compensated patient (sex: M, age: 63, BMI: 25, etiology: instability)

Demographics and OA etiology of the study groups are reported in Table 1. Ankles from the asymptomatic group were statistically younger than ankles from either of the OA groups (*p* > 0.05, *d* = 0.96 (compensated vs asymptomatic), *p* > 0.05, *d* = 0.98 (non-compensated vs asymptomatic)). Group distributions of radiographic measures are as follows: TT 5.7° ± 3.1 and HAA 1.8° ± 5.3 for the compensated group, TT 10.9° ± 5.2 and HAA 23.4° ± 6.0 for the non-compensated group, and TT −0.3° ± 0.6 and HAA −2.1° ± 6.7 for the asymptomatic control group (Fig. 1).

**Table 1:** a) Patient demographic information. (b) Ankle osteoarthritis etiology

	Asymptomatic	Compensated ankle OA	Non-compensated ankle OA
a. Patient characteristics			
Ankles, *n*	26	27	17
Age (y), mean (range)	50 (40–66)	60 (32–80)	59 (40–78)
Body mass index, mean (range)	25 (19–34)	26 (16–30)	28 (25–36)
Sex (%), M/F	27/73	59/41	89/11
Laterality (%), L/R	50/50	41/59	28/72
b. Etiology of ankle OA			
Etiology		n (%)	
Post-traumatic (fracture)		8 (18)	
Post-traumatic (ligamentous)		12 (27)	
Idiopathic (primary)		0 (0)	
Recurring injury		1 (2)	
Instability		21 (48)	
Rheumatoid		1 (2)	
Other (proximal deformity)		1 (2)	
Total		44 (100)

### Morphometric analysis

#### Principal component analysis

**Fig. 2:**
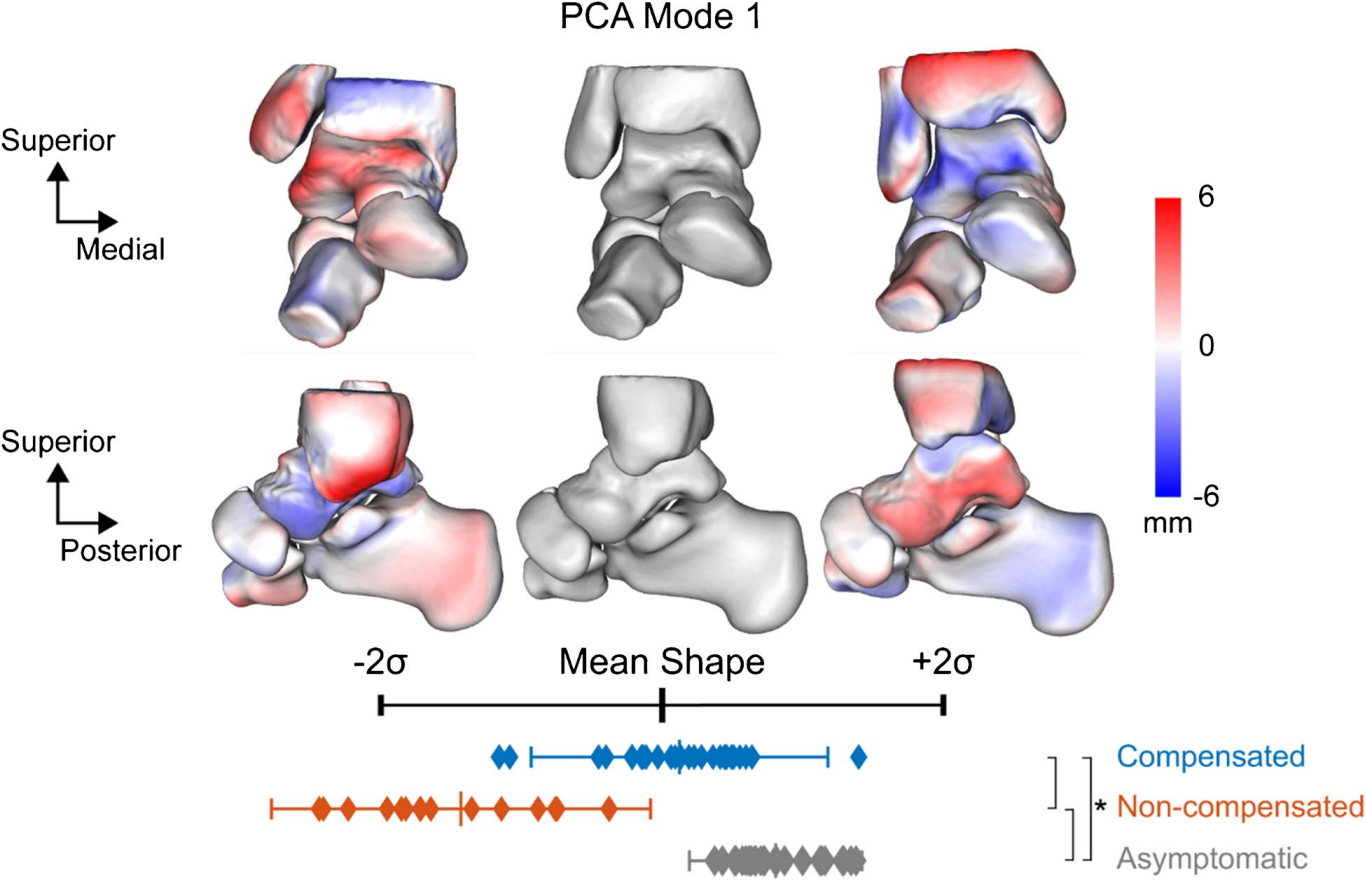
Visualization of the first PCA mode of variation from the SSM. Shape variations denoted at −2σ (left), the mean shape (center), and +2σ (right). Color map indicates the magnitude and direction of deformation, with red regions representing positive deviations (expansion) and blue regions representing negative deviations (contraction) relative to the mean shape. Distribution of subjects along mode one is shown below, categorized into compensated (blue diamonds), non-compensated (orange diamonds), and asymptomatic control (gray diamonds) groups. * indicates statistical difference between groups (*p* ≤ 0.05)

**Fig. 3:**
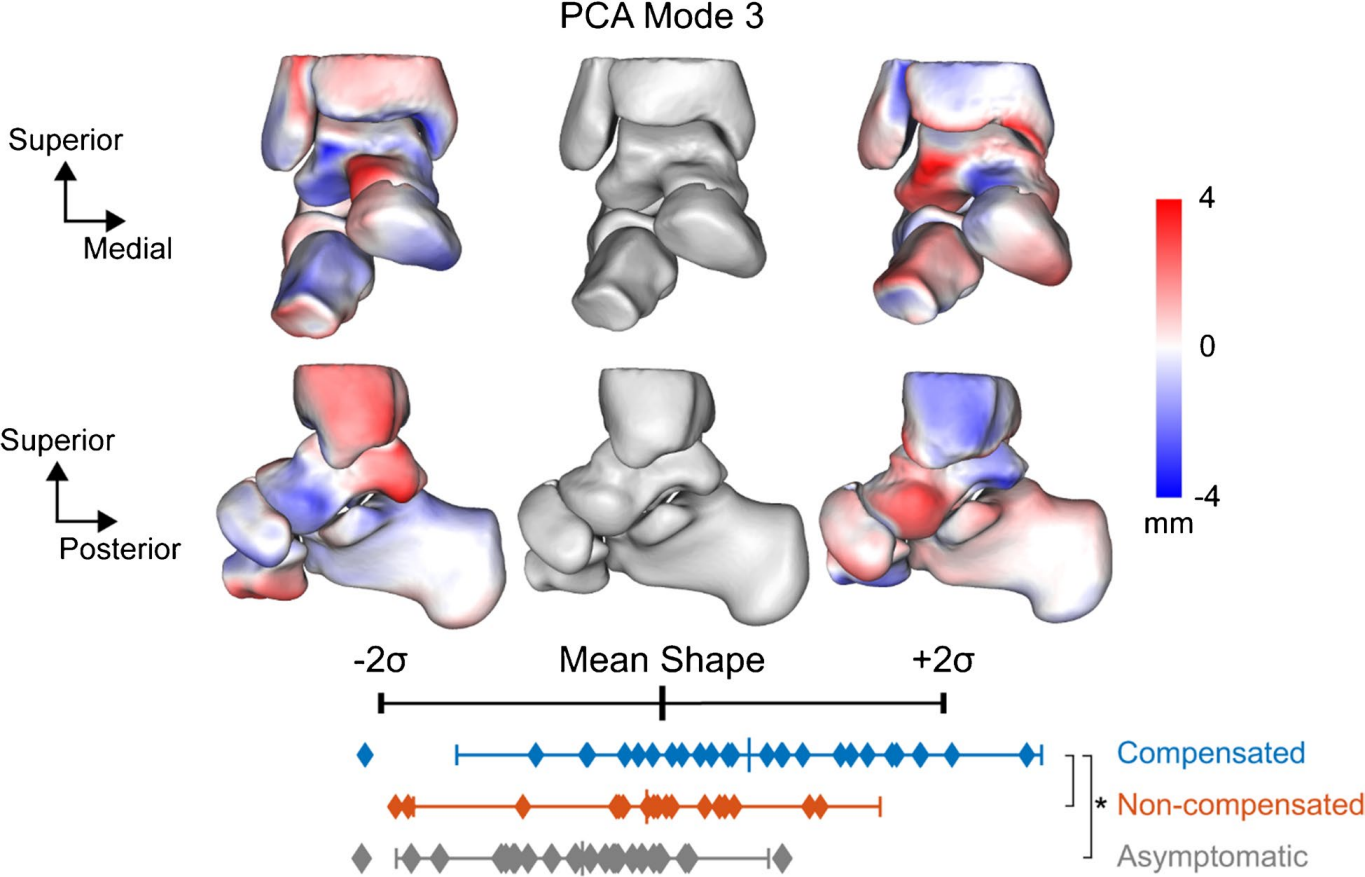
Visualization of the principal component analysis mode three. Shape variations denoted at −2σ (left), the mean shape (center), and +2σ (right). Color map indicates the magnitude and direction of deformation, with red regions representing positive deviations (expansion) and blue regions representing negative deviations (contraction) relative to the mean shape. Distribution of subjects along mode three is shown below, categorized into compensated (blue diamonds), non-compensated (orange diamonds), and asymptomatic control (gray diamonds) groups. * indicates statistical difference between groups (*p* ≤ 0.05)

Eleven PCA modes of variation, which contained 85.8% of the cumulative variance, were retained following a parallel analysis. Group-wise comparisons found a statistical difference in mode 1 between each of the groups. Mode 1 contained 26.6% explained variance and exhibited anatomic variation including distal expansion of the medial malleolus, anterior expansion of the talar dome, abduction of the fibula relative to ankle mortise, and subtle alterations of the calcaneus, navicular, and cuboid. Mean mode 1 scores followed a progressive pattern from non-compensated to compensated to asymptomatic ankles, with significant differences between all groups (Fig. 2) ((compensated mean = 25.2 and 95% confidence interval (CI) = 42.5, non-compensated mean = −291.2 and 95% CI = 70.5, and controls mean = 164.2 and 95% CI = 25.2) [compensated v. non-compensated *p* = 8.9e−4] [compensated v. controls *p* = 1.9e−4] [non-compensated v. controls *p* = 3.6e-12)). Mode 3 contained 10.8% explained variance and a statistical difference was found between the compensated group and both other groups. Anatomic variation in mode 3 included medial/lateral angulation of the talar head, and clockwise/counterclockwise rotation of the cuboideonavicular joint (Fig. 3). Mode 3 demonstrated a different group ordering than mode 1, with mean scores increasing from asymptomatic to non-compensated to compensated ankles ((compensated mean = 83.1 and 95% CI = 55.4, non-compensated mean = −14.9 and 95% CI = 57.4, and controls mean = −76.5 and 95% CI = 36.0) [compensated v. non-compensated *p* = 0.02] [compensated v. controls *p* = 1.3e−5]).

#### Shape and alignment

**Fig. 4:**
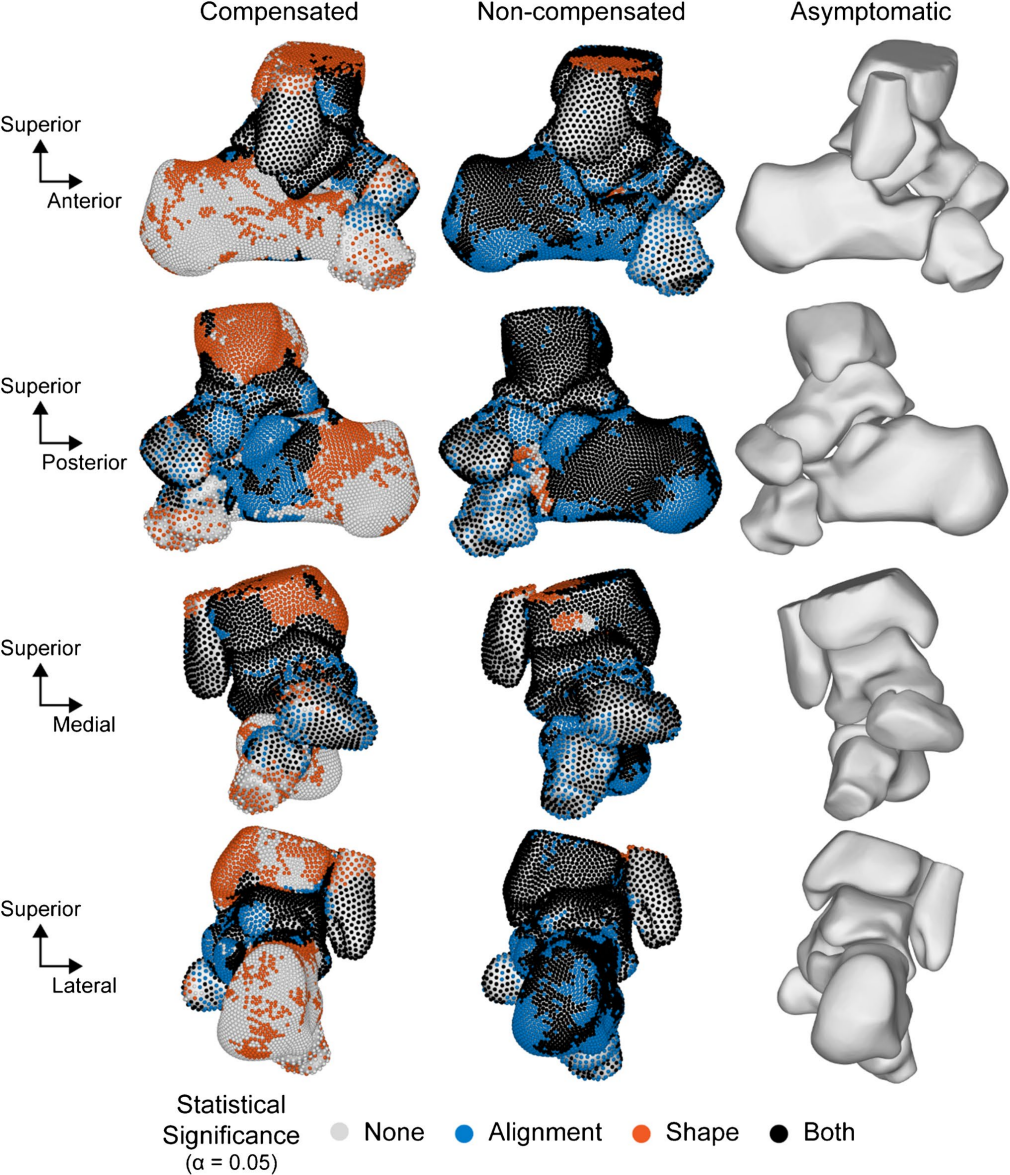
Morphometric comparison of regions of statistical difference (*p* ≤ 0.05) between the compensated (left) and non-compensated (center) varus OA groups and the asymptomatic control group (right). Gray particles indicate regions of no statistical difference. Blue particles show regions of statistical difference solely due to alignment variation. Orange particles denote regions of statistical difference exclusively due to shape variation. Black particles represent regions of statistical difference due to both shape and alignment variation

**Fig. 5:**
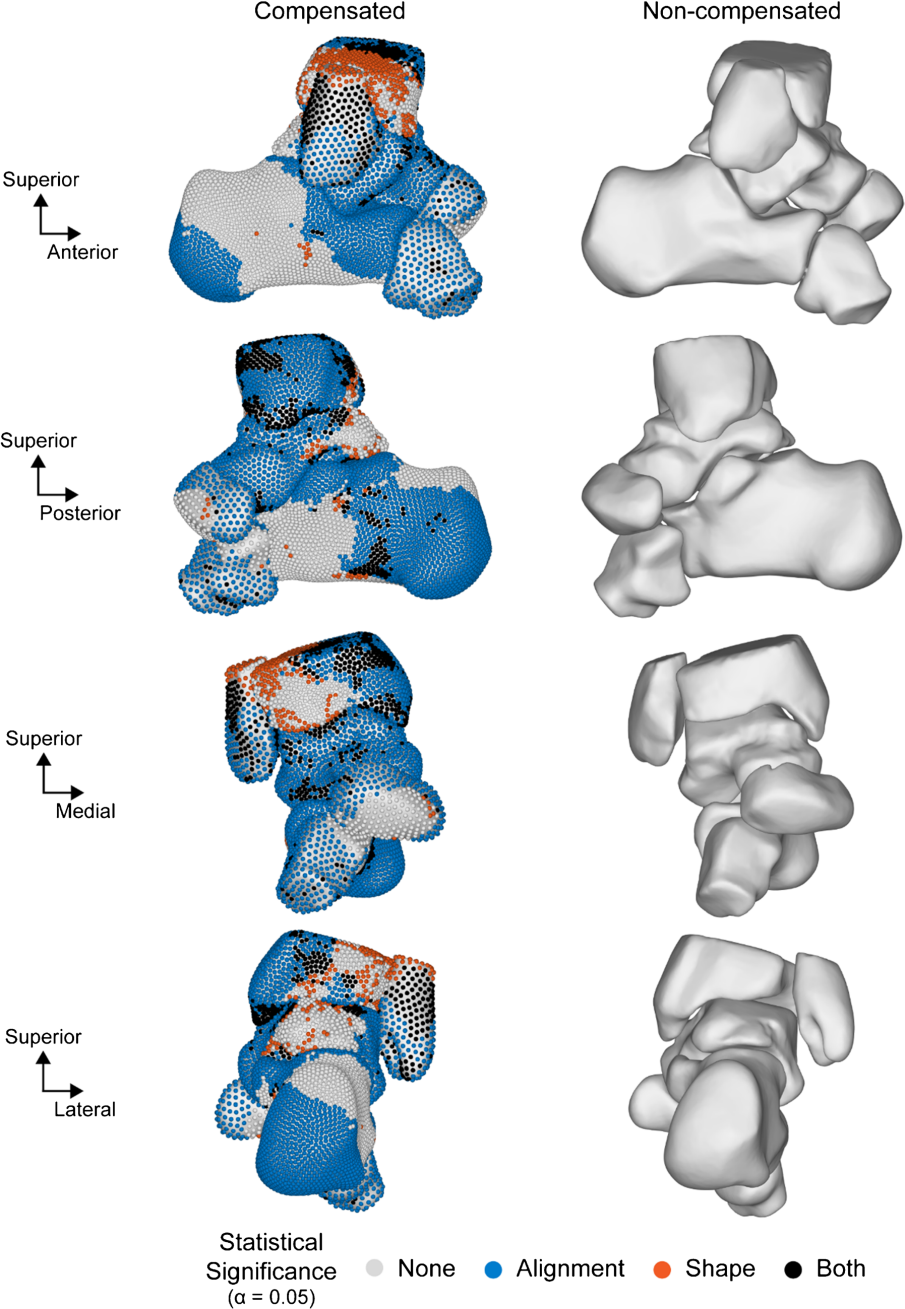
Morphometric comparison of regions of statistical difference (*p* ≤ 0.05) between the compensated (left) and non-compensated (right) varus OA groups. Gray particles indicate regions of no statistical difference. Blue particles show regions of statistical difference solely due to alignment variation. Orange particles denote regions of statistical difference exclusively due to shape variation. Black particles represent regions of statistical difference due to both shape and alignment variation

Morphometric comparison of varus ankle OA groups to the asymptomatic controls found regions of statistical difference. More specifically, compensated compared to controls indicated more isolated shape differences of the distal tibia medial malleolus and medial calcaneus, yet yielded similar regional trends in both alignment and shape and isolated alignment differences as the non-compensated compared to controls. These areas include the bony shape of the peritalar joint complex, with alignment differences of the sustentaculum tali and talar head [compensated v. controls percentages; isolated shape = 26.5%, isolated alignment = 13.7%, both combined = 34.0%, and no significance = 25.8%] [non-compensated v. controls percentages; isolated shape = 4.5%, isolated alignment = 26.8%, both combined = 67.5%, and no significance = 1.2%] (Fig. 4). Morphometric comparisons between the varus ankle OA groups indicated more alignment differences than shape [compensated v. non-compensated percentages; isolated shape = 6.9%, isolated alignment = 55.1%, both combined = 13.1%, and no significance = 24.9%] (Fig. 5).

### Joint measurement analyses

Coverage results are summarized in Table 2. Larger coverage values were observed on the tibial facet of the tibiotalar joint compared to the talar facet within all groups. Smaller coverage values were noted on the calcaneal surface of the subtalar joint compared to the talar surface within all groups. Asymptomatic ankles exhibited smaller tibiotalar and talofibular coverage than compensated and non-compensated ankles. Non-compensated ankles had the largest subtalar joint coverage values followed by compensated ankles; asymptomatic ankles had the smallest subtalar joint coverage values.

**Table 2.**
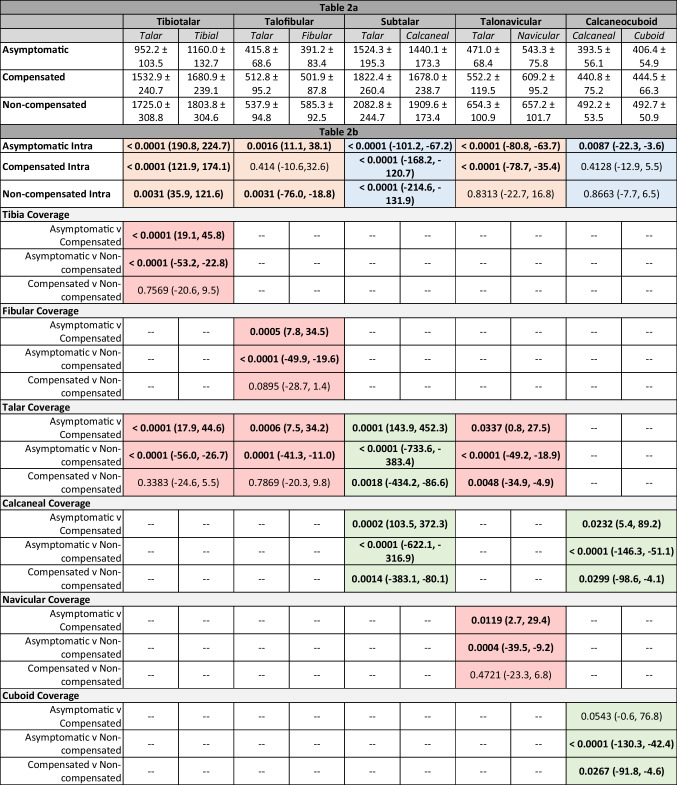
a) Mean ± SD (mm^2^) of articulating joint coverages for asymptomatic, compensated, and non-compensated groups. (b) Statistical analysis of joint coverage values across asymptomatic, compensated, and non-compensated groups reported separately for all seven articulations. Bold values indicate statistically significant difference with an alpha value of 0.05. Each colored cell indicates a different statistical test being performed: blue highlights paired *t*-tests, orange highlights Wilcoxon signed rank tests, green highlights ANOVA with a Tukey’s post hoc test, and red highlights Kruskal-Wallis with a Dunn-Sidak post hoc test

**Fig. 6:**
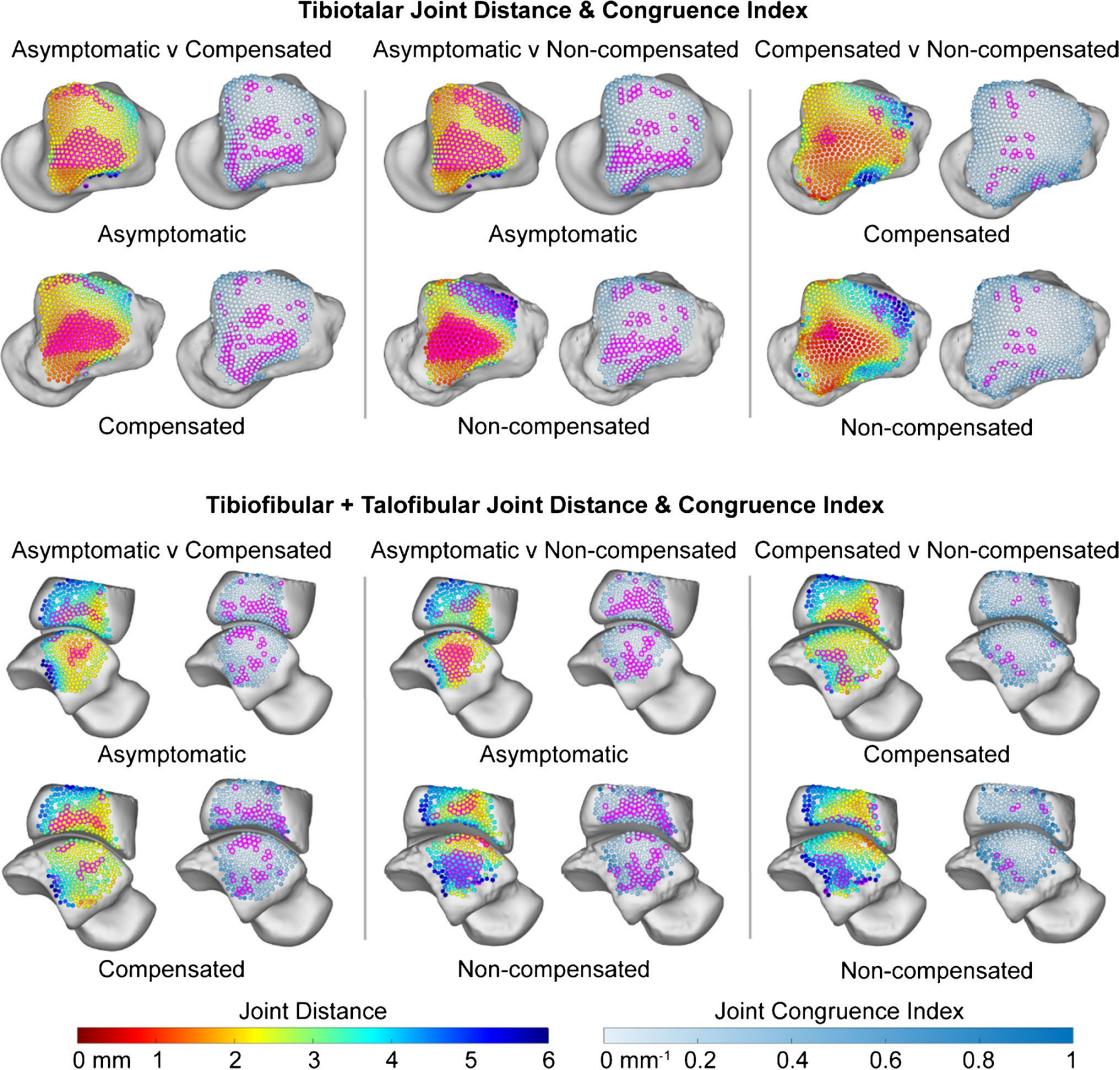
Mean tibiotalar, tibiofibular, and talofibular joint distance and joint congruence indices. Averaged results are visualized for each group on consistent correspondence particle locations. Statistically different values are noted by a pink ring around the correspondence particle in pairwise comparisons

**Fig. 7:**
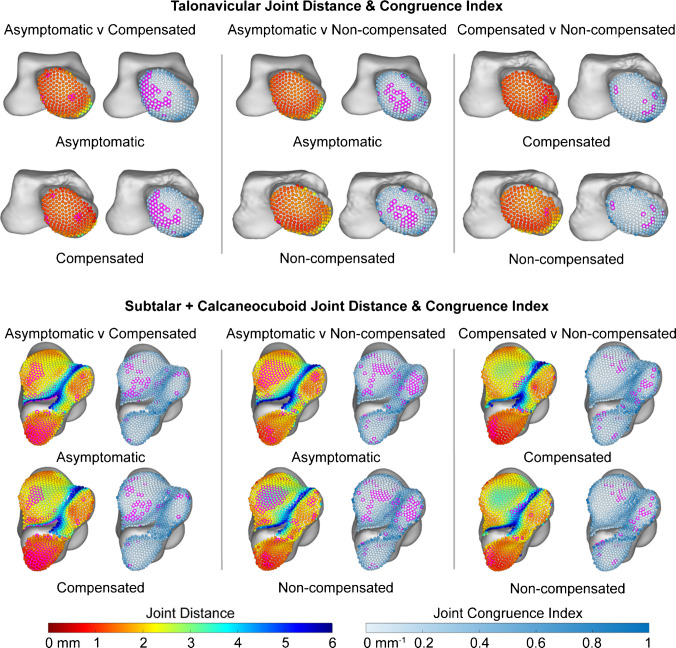
Mean talonavicular, subtalar, and calcaneocuboid joint distance and joint congruence indices. Averaged results are visualized for each group on consistent correspondence particle locations. Statistically different values are noted by a pink ring around the correspondence particle between pairwise comparisons

The talofibular joint of non-compensated ankles exhibited greater distance values than compensated and asymptomatic ankles (Fig. 6). Distance values between the non-compensated and compensated tibiotalar joints were comparable, and greater joint distances were observed in the asymptomatic tibiotalar joints. Smaller joint distance values were noted between the compensated and non-compensated groups compared to the asymptomatic group across the posterior facet of the subtalar joint. For the anterior facet of the subtalar joint, greater joint distance values were seen in the asymptomatic and compensated ankles when compared to non-compensated ankles (Fig. 7). Talonavicular distance values were comparable between all groups. Smaller distance values were observed in calcaneocuboid joints of compensated ankles compared to asymptomatic and non-compensated ankles (Fig. 7).

Greater congruence was observed in the tibiofibular and talofibular joints of asymptomatic ankles compared to compensated and non-compensated ankles (Fig. 6). Talofibular joints in compensated ankles showed greater congruence than non-compensated ankles (Fig. 6). There was greater congruence in the middle facet of the subtalar joint of non-compensated ankles compared to asymptomatic and compensated ankles (Fig. 7). Calcaneocuboid congruence values were comparable between all groups (Fig. 7).

## Discussion

Peritalar joint compensation may contribute to the preservation of the ankle joint and protect against further deformity in cases of asymmetric varus ankle osteoarthritis [4]. The aim of this study was to evaluate peritalar bone morphology and alignment in two groups with advanced varus ankle OA with either a neutral (compensated) or varus (non-compensated) hindfoot alignment as compared to a third group of asymptomatic individuals. Morphologic and alignment differences between the mean compensated and mean non-compensated bones include [1] distance between medial malleolus and sustentaculum tali, [2] orientation of the midtarsal joint complex, [3] talofibular relationship, and [4] internal rotation of the talus in the non-compensated ankles compared to compensated ankles (Fig. 5). These findings emphasize the impact of peritalar compensation on the morphometrics of osteoarthritic ankles, presenting a comprehensive quantitative analysis between these patient populations. Our observations agree with previous 2D findings in the literature that have demonstrated differences in hindfoot alignment and talar positioning between compensated and non-compensated varus ankles, and we conclude that there are consistent 3D differences between compensated and non-compensated ankles demanding careful management and correction [7, 15, 22, 38].

### Morphologic variation and alignment

Bony morphology has been shown to be a potential risk factor in OA development [39]. An article by Manter determined that the orientation of the midtarsal joint is reliant on the position of the subtalar joint, and we see that the more supinated subtalar joint in non-compensated ankles corresponds to a more stable midtarsal orientation [40]. Combining morphology and alignment provides comprehensive insights into differences between clinical presentations of advanced varus ankle OA. The morphometric contrast between the compensated and non-compensated shows predominantly alignment differences rather than shape or combined differences. Interestingly, we see combined differences near the proximal attachment of the deltoid ligament at the medial malleolus. Comparing the varus groups to the asymptomatic controls displayed combined shape and alignment differences as well as a considerable amount of purely shape differences across the tibia, calcaneus, and cuboid. The differences noted within the sustentaculum tali appear to be mostly alignment oriented rather than shape. Differences in directional patterns between PCA scores among groups suggest that modes 1 and 3 represent distinct morphologic features associated with global varus deformity and peritalar compensatory adaptations, respectively. The distinct morphometric profiles observed between compensated and non-compensated varus ankle OA suggest tailored therapeutic strategies could be more effective. For instance, interventions aimed at correcting alignment might be sufficient for non-compensated patients, whereas compensated patients may require treatments addressing both shape and alignment abnormalities. Realignment procedures such as a calcaneal osteotomy may necessitate further attention due to the calcaneal morphological differences we present (Figs. 4 and 5).

Based on the variation in bony morphology and alignment across the six bones we analyzed, the most logical explanation is that there are abnormal ligamentous structural differences between compensated and non-compensated ankles. Such differences could be owed to anatomic variation insinuating the need to investigate peritalar compensation with image modalities sensitive to soft tissue structures. These observations underscore the importance of considering both shape and alignment in clinical evaluations and interventions.

### Joint measurement analyses

Joint analyses are a complex but crucial effort in understanding skeletal deformities and their impact on neighboring joint mechanics and quantifying disease condition [41]. The larger joint distance values for the talofibular joint of non-compensated ankles confirm the differences we see in morphology compared to asymptomatic and compensated ankles. Prior literature has noted that tibiotalar joint congruence in the case of varus ankle OA is lost giving rise to excessive wear of the medial tibiotalar joint [42]. Our findings support this pattern when comparing joint distance and joint congruence index measurements between the three groups analyzed. The anterior facet of the subtalar joint exhibited smaller distance values as well as increased congruence index in non-compensated ankles than in asymptomatic and compensated ankles, indicative of more severe bone approximation seen in advanced OA. Combining these results with the more pronounced sustentaculum tali in non-compensated ankles, noted in the morphology, we postulate that the anteromedial facet of the subtalar joint plays a vital role in varus hindfoot compensation as it may provide an additional bony prominence for joint support. Difference in the congruence index of the talonavicular joint between groups may be due to the differences in morphology and joint orientation among these groups. The lateral border of the cuboidal articular surface of compensated calcanei exhibited noticeable impingement compared to non-compensated calcanei, potentially due to calcaneocuboid joint orientation. The differences in coverage area between the three groups we analyzed could be explained by severe subluxation and/or differences in ankle stability between the three groups. It is possible that a compensatory mechanism is the normal reaction of the peritalar joints in cases of progressive varus deformity at the tibiotalar joint (deformity progression from superior to inferior). In non-compensated ankles, however, a subtle cavovarus deformity might be present initially (deformity progression from inferior to superior). Although medical history may include several ankle sprains in both patient groups, the underlying etiology may be different. Also, osteophytes and malalignment in the OA groups could account for the larger coverage values seen when compared to the asymptomatic group.

### Limitations

A limitation of the study is the variable amount of tibial shaft present in the WBCT scans; a large field of view should be used when imaging patients in a WBCT scanner to measure tibial axes consistently. Second, our retrospective study lacked a prospective inclusion of age-matched asymptomatic controls with large age ranges for OA patients that may have an effect on our findings. Sex distribution differed between the groups in our study; however, prior work by Moore et al. showed that sex-based ankle bone morphologic differences are subtle. Therefore, this discrepancy is unlikely to have substantially influenced our results [43]. Additionally, our assessment is of patients in a static state; therefore, we may not fully capture the functional consequences of the observed morphologic differences between our patient groups during dynamic activities of daily living. Our joint measures reflect subchondral bone-to-bone surfaces thereby neglecting cartilaginous contributions to joint analyses. Distinguishing native articular margins from osteophytic bone is challenging, and as such, osteophytes may have contributed to some of the measured articular coverage values. As a retrospective single time point study, we are not able to quantify disease progression and development over time. Longitudinal studies would enable quantification of OA progression of morphologic changes over time.

### Clinical implications

Understanding the bony morphologic differences in patients with advanced varus ankle OA can aid clinicians in accurately diagnosing and staging disease progression. While standard weight-bearing radiographs remain the primary modality for evaluating coronal alignment, our findings demonstrate that radiographically similar alignment patterns may be associated with distinct underlying osseous morphologies. Moreover, identifying a patient’s unique bony abnormalities and joint alignments can help guide individualized approaches of treatment strategies leading to better outcomes and improved management of advanced ankle OA.

Selection of surgical procedure and treatment of advanced varus ankle OA may need to be adapted in the presence or absence of peritalar compensation to ensure natural bilateral hindfoot alignment following operative treatment as persistent malalignment following surgical treatment may lead to exacerbation of OA progression [44]. In cases of compensated varus deformity, anterior approach total ankle arthroplasty allows for correction of the varus malalignment while preserving the lateral collateral ligaments. However, in non-compensated varus cases, lateral approach total ankle arthroplasty may be preferred as it provides better access and visualization of the lateral aspect of the joint and allows for direct correction of the varus alignment and lateral ligament reconstruction. Advanced varus ankle OA patients electing an ankle arthrodesis procedure may further exacerbate joint relationships distal to the tibiotalar joint especially in compensated varus OA patients. It is difficult to say if hindfoot alignment was corrected in the non-compensated group either via arthroplasty or arthrodesis, if the noted differences in orientation of the cuboid and navicular would return to natural alignment.

The WBCT analyses in this study are not intended to advocate for routine preoperative WBCT imaging in all patients with varus ankle OA. Rather, these findings establish a morphologic basis for compensated and non-compensated alignment patterns that may not be fully captured on plain films alone. In current practice, clinicians can continue to use weight-bearing radiographs to identify global alignment and compensatory patterns, reserving WBCT imaging as a targeted adjunct in select cases when detailed assessment of osseous morphology may influence surgical planning. Future research should investigate postoperative outcomes to improve our understanding of preoperative surgical planning and optimize patient outcomes. Our study illustrates the importance of using 3D analyses to better evaluate clinical presentations of varus ankle OA.

## Data Availability

Data may be made available by the authors upon request.
